# Neutralization-based seroprevalence of Toscana virus and sandfly fever Sicilian virus in dogs in the Republic of Kosovo

**DOI:** 10.1186/s13071-025-06681-7

**Published:** 2025-02-10

**Authors:** Betim Xhekaj, Elif Kurum, Jovana Stefanovska, Aleksandar Cvetkovikj, Kurtesh Sherifi, Agim Rexhepi, Remi Charrel, Edwin Kniha, Nazli Ayhan

**Affiliations:** 1 Faculty of Agriculture and Veterinary, University of Prishtina “Hasan Prishtina”, Bulevardi “Bill Clinton”, 10000 Pristina, Kosovo; 2 Unité des Virus Émergents (UVE: Aix-Marseille Univ, Università di Corsica, IRD 190, Inserm 1207, IRBA), Marseille, France; 3 Faculty of Veterinary Medicine-Skopje, Ss. Cyril and Methodius University in Skopje, Lazar Pop-Trajkov 5-7, Skopje, 1000 North Macedonia; 4 Center for Pathophysiology, Infectiology and Immunology, Institute of Specific Prophylaxis and Tropical Medicine, Medical University Vienna, Kinderspitalgasse 15, 1090 Vienna, Austria; 5 National Reference Center for Arboviruses, Inserm-IRBA, Marseille, France

**Keywords:** *Phlebovirus*, Toscana virus, Sand fly fever, Seroneutralization assay, Balkan, Stray dogs

## Abstract

**Background:**

Phlebotomine sand flies are the key vectors for phleboviruses (order Hareavirales and family Phenuiviridae), of which some are associated with febrile diseases and nervous system infections. In the Mediterranean Basin, Toscana virus (TOSV; *Phlebovirus toscanaense*) and sandfly fever Sicilian viruses (SFSV; *Phlebovirus siciliaense*) are important human pathogens, and their endemicity has been known for decades, particularly in the Balkan countries. While the circulation of both viruses is highly evident among humans and livestock in the Central Balkan country Kosovo, data from companion animals are scarce; however, it might help to further assess the distribution of both viruses in the country.

**Methods:**

Sera of dogs from all seven districts of Kosovo were screened for TOSV and SFSV antibodies by seroneutralization assays.

**Results:**

Altogether, 45 of 288 (15.6%) samples showed anti-*Phlebovirus* antibodies, of which 36 (12.5%) were against TOSV, 11 (3.8%) were against SFSV, and 2 (0.7%) were positive for antibodies against both viruses.

**Conclusions:**

*Phlebovirus* seroprevalence was observed in all seven districts of the country, generally being higher for TOSV compared with SFSV. Our study presents the first assessment of neutralization-based seroprevalence of two medically important phleboviruses among dogs in the Republic of Kosovo. Although healthy dogs are unsusceptible to *Phlebovirus* infection, dogs with leishmaniasis can be potential amplifying hosts. Given the high number of stray dogs, frequent uncontrolled spreading of phleboviruses in dogs, and potential spillover in populated regions of the country, these findings should be taken into consideration.

**Graphical Abstract:**

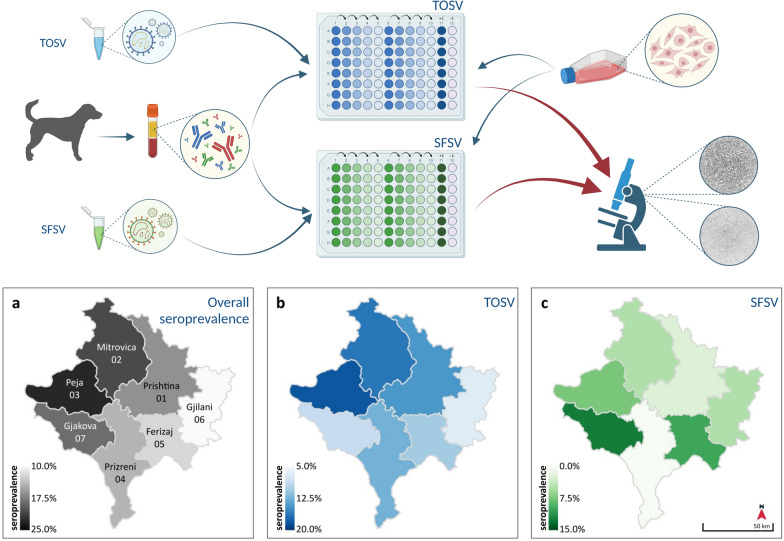

**Supplementary Information:**

The online version contains supplementary material available at 10.1186/s13071-025-06681-7.

## Background

Phleboviruses (order Hareavirales and family Phenuiviridae) are negative-sense trisegmented RNA viruses, of which phlebotomine sand flies (Diptera, Psychodidae, Phlebotominae) are the principal vectors. Currently, 67 species have been officially assigned by the International Committee of Taxonomy of Viruses (ICTV, https://ictv.global/taxonomy). In Eurasia and Africa, Toscana virus (TOSV; *Phlebovirus toscanaense*), sand fly fever Naples virus (SFNV; *Phlebovirus napoliense*), and sand fly fever Sicilian virus (SFSV; *Phlebovirus siciliaense*) were the three sand fly-borne viruses that were recognized human pathogens. Toscana virus (TOSV) is present in the Mediterranean Basin. Sand fly fever Sicilian virus (SFSV) and sand fly fever Naples virus (SFNV) are more widely distributed in Europe, Africa, and Asia [1]. The majority of *Phlebovirus* infections remain asymptomatic, but can result in febrile illness with sudden high fever, headache, photophobia, malaise, and retro-orbital pain; the symptoms usually decline after a few days. The disease is referred to as “sand fly fever” [2]. Noteworthy, TOSV shows a strong neurotropism and may cause central and peripheral nervous system infections, such as meningitis and encephalitis [3].

While the endemicity of sand fly fever in the Balkans has been known for decades, other phleboviruses have been discovered more recently: Adria virus, Balkan virus, and Drin virus were recently described in Albania [4–6], Zaba virus in Croatia, and Bregalaka virus in North Macedonia [7].

In the Republic of Kosovo, the circulation of TOSV and SFSV is supported by the detection of high antibody levels in livestock [8] and Austrian soldiers serving in Kosovo [9]. To date, no data on the circulation of these two viruses in sand flies are available; however, the distribution and high abundance of the potential vectors, *Phlebotomus neglectus* and *Phlebotomus perfiliewi*, were described recently in all seven regions of the country [10–13].

No reservoir hosts have been clearly defined for phleboviruses, and competent sand fly species might act as main reservoirs in the viral cycle, which may be boosted with blood meals from viremic vertebrate hosts [14]. For some phleboviruses, such as TOSV, dogs have been suspected as important reservoir hosts [15]. Although the reservoir role of healthy dogs is not supported by experimental evidence [16], it has been reported that dogs presenting with active zoonotic visceral leishmaniasis (ZVL) display viremia, which allows the infection of naïve sand flies under natural conditions [17]. Regardless of the role of dogs in the natural cycle of such viruses, they are good sentinels to address virus circulation.

Our study aimed to analyze TOSV and SFSV seroprevalence in dogs from all seven regions of Kosovo to assess the circulation of both viruses in the country.

## Methods

### Dog samples and sample size

Serum samples were collected from dogs in the frame of a *Leishmania* seroprevalence study in Kosovo [18]. Samples originated from dogs in private households, stray dogs (kept in shelters), and shepherd dogs. The majority of dogs were kept outdoors exclusively. All samples were collected following the basic ethical principles and were marked with the name of the dog or chip number, location, age, breed, sex, and health status.

A total of 288 dogs were included, comprising 147 females and 141 males from all seven districts, collected over a 1-year period between summer 2021 and spring 2022. The mean age was 3.9 years (standard deviation, SD: 2.7), with the youngest dog being 4 months and the oldest 16 years. A total of 112 (38.9%) were of a specified breed and 169 (61.1%) were of mixed breed. Of all the dogs, 246 (85.4%) were classified as healthy and 42 (14.6%) as disrupted (random pathology unrelated to canine leishmaniasis such as dermatitis, arthritis, tumor, or vasculitis). In total, 50 samples originated from Prishtina district (01), 40 from Mitrovica (02), 38 from Peja (03), 40 from Prizreni (04), 41 from Ferizaj (05), 40 from Gjilani (06), and 39 from Gjakova (07).

### Seroneutralization assay

Dog sera were heat-inactivated at 56 °C for 30 min, then diluted from 1:10 to 1:80 and mixed 1:1 with 100 TCID50 of TOSV (MRS2014-44725) or SFSV (Sabin) in 96-well plates. After 1 h of incubation at 37 °C, 100 μL of a Vero E6 cell suspension (5 × 10^5^ cells/mL) was added, resulting in final serum dilutions ranging from 1:20 to 1:160. Negative and positive controls were included in each microplate. After 5 days, cytopathic effect (CPE) was examined, with neutralization (NT) titers recorded at 20, 40, 80, and 160. A seropositivity cutoff was set at a titer of ≥ 40.

### Statistical analysis and mapping of prevalences

Data were prepared with Microsoft Excel for Mac and analyzed with RStudio for Mac [19]. Categorical data (age, breed, district, health status, *Leishmania* seroprevalence, and sex) were analyzed with Fisher’s exact test, using overall prevalence, TOSV, and SFSV prevalence as predictor variables. Odds ratios (OR) with exact 95% confidence intervals (CI) were estimated. On a municipality level, we refrained from statistical analysis owing to the partially low number of available samples. A two-sided *p*-value < 0.05 was considered statistically significant. Prevalence was mapped with QGIS [20] using first-level administrative divisions of Kosovo (year 2015) taken from https://earthworks.stanford.edu/catalog/stanford-zh532mm5047.

### Results

### Phlebovirus seroprevalence and antibody titers

Altogether, 45 of 288 (15.6%) samples showed neutralizing antibodies (NT-Ab) against either TOSV or SFSV or both; 36 (12.5%) sera contained TOSV NT-Ab, 11 (3.8%) sera contained SFSV NT-Ab, and 2 (0.7%) sera contained NT-Ab against the two viruses. NT titers ranged from 1:40 to 1:160 for TOSV and from 1:40 to 1:80 for SFSV (Table 1).

**Table 1:** Phlebovirus seroprevalence and antibody titer levels

Antibody titer	TOSV	SFSV
1:40	26 (9.0%)	10 (3.5%)
1:80	8 (2.8%)	1 (0.3%)
1:160	2 (0.7%)	–
Total (*n* = 288)	36 (12.5%)	11 (3.8%)

Generally, no significant differences between seroprevalence rates and analyzed factors were observed (Table 2). Overall seroprevalence, as well as individual TOSV and SFSV, were all higher in dogs with a normal health status compared with those with a disrupted health status. Similarly, specified breed dogs showed higher seroprevalence rates for both viruses compared with mixed breeds. Two dogs showed *Leishmania* Ab as well as TOSV NT-Ab; however, dogs that had previously tested positive for *Leishmania* Ab did not exhibit significantly higher rates. Also, there was no clear trend in relation to the age of the dogs. For TOSV, the highest rates were detected in age groups 0–2 and 3–4 years, while for SFSV prevalence rates were highest in dogs aged more than 8 years and 3–4 years (Table 2).

**Table 2:** Seroprevalence associated with different factors (*P* = *p*-value). The factor *Leishmania* indicates previous *Leishmania* seroprevalence

Parameter (*n*)	Overall	OR (95% CI), *P*	TOSV	OR (95% CI), *P*	SFSV	OR (95% CI), *P*
Sex						
Female (147)	14.3%	Reference	10.9%	Reference	4.1%	Reference
Male (141)	17.0%	1.3 (0.6–2.5), 0.6	14.2%	1.4 (0.6–2.9), 0.5	3.6%	0.9 (0.2–3.5), 1
Health status						
Disrupted (42)	11.9%	Reference	11.9%	Reference	–	Reference
Normal (246)	16.3%	1.4 (0.5–5.0), 0.7	12.6%	1.1 (0.6–3.7), 1	4.5%	–
Breed						
Mixed (177)	14.1%	Reference	12.4%	Reference	2.3%	Reference
Specified (111)	18.0%	1.3 (0.7–2.7), 0.4	12.6%	1.0 (0.5–2.2), 1	6.3%	2.9 (0.7–13.8), 0.1
*Leishmania*						
Negative (275)	15.6%	Reference	12.4%	Reference	4.7%	Reference
Positive (13)	15.4%	1.0 (0.1–4.7), 1	15.4%	1.3 (0.1–6.3), 0.7	–	–
Age, years						
0–2 years (42)	19.1%	Reference	16.7%	Reference	2.4%	Reference
2–3 years (63)	14.3%	0.7 (0.2–2.3), 0.6	11.1%	0.6 (0.2–2.3), 0.6	4.8%	2.0 (0.2–110.1), 0.6
3–4 years (60)	20.0%	1.1 (0.4–3.4), 1	16.7%	1 (0.3–3.4), 1	5.0%	2.1 (0.2–115.9), 0.6
4–6 years (65)	12.3%	0.6 (0.2–2.0), 0.4	9.2%	0.5 (0.1–1.9), 0.4	3.1%	1.3 (0.1–78.6), 1
6–8 years (26)	11.5%	0.6 (0.1–2.7), 0.5	11.5%	0.7 (0.1–3.3), 0.7	–	–
> 8 years (32)	15.6%	0.8 (0.2–3.1), 0.8	9.4%	0.5 (0.1–2.5), 0.5	6.3%	2.7 (0.1–164.9), 0.6

### Prevalence by district

**Fig. 1:**
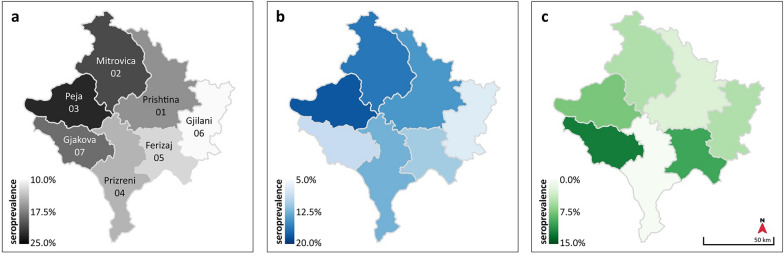
Phlebovirus seroprevalence by district in the Republic of Kosovo. Overall seroprevalence (**a**), TOSV (**b**), and SFSV (**c**)

Overall, nonsignificant seroprevalence rates were highest in Peja (21.1%) and lowest in Gjilani (10.0%). For TOSV, rates were highest in Peja (18.4%) and Mitrovica (17.5%) and lowest in Gjakova (7.7%) and Gjilani (7.5%). SFSV rates were highest in Gjakova (12.8%) and considerably lower in all other districts (maximum of 4.9%). No SFSV NT-Ab were detected in samples originating from Prizreni (Table 3, Fig. 1). Dual reactivity against TOSV and SFSV was detected in two samples originating from Mitrovica and Peja. Owing to the stringency of the NT assay, the two samples that exhibited double reactivity must be interpreted as having been collected from dogs who were infected independently with TOSV and SFSV, and not as a cross-reactivity of the NT assays.

**Table 3:** Phlebovirus seroprevalence by district. Districts with lowest seroprevalence were used as references for statistical comparison

ID	District (samples)	Total	TOSV	SFSV
		Nb^*^ ≥ 40 (%)	Nb^*^ ≥ 40 (%)	Nb ^*^ ≥ 40 (%)
		OR (95% CI), *P*	OR (95% CI), *P*	OR (95% CI), *P*
01	Prishtina (*n* = 50)	8 (16.0%)	7 (14.0%)	1 (2.0%)
		1.7 (0.4–8.4), 0.5	2.0 (0.4–12.8), 0.5	Reference
02	Mitrovica (*n* = 40)	8 (20.0%)	7 (17.5%)	1 (2.5%)
		2.2 (0.5–11.1), 0.4	2.6 (0.5–16.8), 0.3	1.3 (0.0–100), 1
03	Peja (*n* = 38)	8 (21.1%)	7 (18.4%)	1 (2.6%)
		2.4 (0.6–11.9), 0.2	2.8 (0.6–17.9), 0.2	1.3 (0.0–106), 1
04	Prizreni (*n* = 40)	5 (12.5%)	5 (12.5%)	0 (0.0%)
		1.3 (0.3–7.0), 1	1.8 (0.3–12.1), 0.7	–
05	Ferizaj (*n* = 41)	5 (12.2%)	4 (9.8%)	2 (4.9%)
		1.3 (0.3–6.8), 1	1.3 (0.2–9.7), 1	2.5 (0–151), 0.6
06	Gjilani (*n* = 40)	4 (10.0%)	3 (7.5%)	1 (2.5%)
		Reference	Reference	1.3 (0–100), 1
07	Gjakova (*n* = 39)	7 (17.9%)	3 (7.7%)	5 (12.8%)
		1.9 (0.5–10.0), 0.5	1.0 (0.1–8.2), 1	7 (0.7–347), 0.08

* Nb: Total sample count.

Samples were available from 29 of 38 municipalities. Overall, both TOSV and SFSV seroprevalence ranged from 0% to 50%. Owing to the low number of samples in some municipalities, we refrained from detailed analysis (Additional file 1: Supplementary Table 1). Prevalence rates by municipality are presented in Additional file 2: Supplementary Fig. 1, in an illustration of *Phlebovirus* seroprevalence.

## Discussion

Our study presents the first assessment of neutralization-based seroprevalence of two medically important phleboviruses among dogs (as sentinels) in the Republic of Kosovo. While indicating the circulation of both viruses among dogs, we found marked seroprevalence differences between TOSV and SFSV.

Generally, the rates of TOSV NT-Ab were more than three times higher than SFSV NT-Ab, demonstrating that the dogs’ exposure to TOSV is much higher than to SFSV. This is probably also the case for the human population, but this assumption merits confirmation through a purposely designed study. The observation of high titers denotes either a strong infection, repeated exposure to the virus, or both.

For TOSV, the 12.5% positivity rate is in agreement with data reported in previous studies of domestic animals in TOSV-endemic countries such as France (3.9% in Corsica) [21], Portugal (6.8%) [22], or Spain (36.2%) [23]. Interestingly, a single previous serosurvey among cattle and sheep in Kosovo showed a lower prevalence of 4.7% compared with our study, despite using the same methodology [8]. However, Ayhan et al. [8] analyzed samples collected in 2013 on a municipality level (38 municipalities) mostly in the western part of the country from different animal species (cattle and sheep). These differences in sampling year, geographic focus, and host species could significantly impact the observed prevalence, as they may influence the hosts’ immune responses to viral infections or reflect variations in the virus’ circulation frequency over time. In addition, it should be underlined that positivity in sentinel animals such as dogs, cattle, or sheep should be analyzed qualitatively (presence of the virus) and not quantitatively (serological positivity rate) to assess their involvement in the transmission cycle. In their study [8], seroprevalence rates varied from 0 to 11%, while our samples were primarily analyzed by administrative districts (7 districts), ranging from 7.5% to 18.4%. While we also present data by municipality, it must be taken into account that the sample size was too small for detailed analyses, but prevalence by municipality might serve as baseline data for further targeted surveillance studies on vector species. Possibly, transmission cycles may have shifted in the last 10 years in the country.

Contrary to TOSV, our detected SFSV seroprevalence was much lower compared with other studies that show 50.8% in dogs in Portugal [22] and 53.3% and 27.5% in cows and sheep, respectively, in Saudi Arabia [24], or 53.4% in cattle and sheep from Kosovo [8]. While confirming SFSV circulation among dogs in six of the seven districts in Kosovo, the prevalence was generally very low and only high (12.5%) in the western district Gjakova. Again, we would like to stress that these differences cannot be attributed to technical reasons since the same methodology was applied in most of the referenced studies that were conducted in our French laboratory; the only exception is the study by Al-numaani et al. [24] that was performed in Saudi Arabia with the same protocol and with the same virus strains. This applies to both TOSV and SFSV data.

Regional differences of TOSV and SFSV seroprevalence should be further discussed taking vector presence and abundance into account. Initially, *Phlebotomus perniciosus*/*Phlebotomus perfiliewi* and *Phlebotomus papatasi* were identified as the principal vectors of TOSV and SFSV, respectively; however, newer studies highly indicate that other sand fly species might be involved in their transmission [2]. Several recent entomological surveys addressed the sand fly distribution, diversity, and abundance in Kosovo [11, 25, 26]. While nine species are endemic, *Phlebotomus neglectus* and *Phlebotomus perfiliewi* are the two most widely distributed and the most abundant species, highlighting their potential involvement in TOSV transmission [25]. Although SFSV is linked to *Phlebotomus papatasi*, this species has only been found in low numbers at a few locations, clearly indicating that other sand fly species may play a role as vectors for SFSV in Kosovo. In Turkey, *Phlebotomus major* sensu lato is the suspected principal vector of the recently identified sandfly fever Sicilian virus variant, sandfly fever Turkey virus [27]. In Greece, a closely related virus, Corfou virus, has been isolated from *Phlebotomus neglectus* [28]. Thus, the role of *Phlebotomus neglectus* and *Phlebotomus perfiliewi* as vectors of SFSV should be assessed in further experiments. The highest prevalence rates in our study were observed in the western districts of Peja for TOSV and Gjakova for SFSV, which coincide with the highest sand fly abundance and match the area with the highest climatic suitability values for sand fly presence [26]. Additionally, blood meal analysis from entomological surveys in Kosovo revealed *Phlebotomus neglectus* and *Phlebotomus perfiliewi* to be multihost-feeding, preferring cattle and sheep as host animals, but also feeding on dogs and humans on fewer occasions, thereby potentially promoting occasional spillover between hosts [11]. Of note, both sand fly species are likely involved in the transmission of *Leishmania infantum* in Kosovo, which is also endemic in all seven districts [10, 18].

Similar to other studies (e.g., in Algeria) [29], we did not statistically observe any clinical relevance of *Phlebovirus* infections in dogs. Some *Phlebovirus* infections are associated with symptoms in host animals, such as deadly hemorrhagic fevers in cats infected with severe fever with thrombocytopenia virus (SFTSV) [30] or miscarriage in livestock infected with Rift Valley fever virus (RVFV) [31]. Additionally, dogs artificially infected by TOSV and SFSV did not show any clinical symptoms [16]. Despite the high medical relevance of TOSV and SFSV in humans, the veterinary relevance of these two viruses is unknown.

We did not observe a statistical interaction between *Phlebovirus* and *Leishmania infantum* seropositivity in our sample population, which might be explained by the potentially focal appearance of these pathogens; however, as we detected two dogs positive for anti-*Leishmania* as well as anti-TOSV antibodies, coinfections might result in a different clinical outcome. However, Dincer et al. (2015) did not observe any clinical connection between TOSV and *Leishmania infantum* infections in dogs; a recent study demonstrated that colonized *Phlebotomus perniciosus* sand flies that fed on dogs with ZVL were found to be infected with TOSV, which may indicate the potential of dogs with leishmaniasis playing a role as reservoir for TOSV in Tunisia [17]. Additionally, another recent study by Heirwegh et al. (2021) [32] demonstrated a significantly higher parasite burden in *Leishmania major* and SFSV coinfected mice compared with individuals with sole *Leishmania major* infections, indicating a need for further studies.

## Conclusions

In our study, we clearly demonstrated the endemicity of TOSV and SFSV in Kosovo, two medically important phleboviruses. Considering the high number of stray dogs in Kosovo and the fact that dogs are mainly kept outside, living in close contact might facilitate spillovers to the human population through abundant vector species.

## Supplementary Information

Additional file 1: Table S1. Phlebovirus seroprevalence by municipality in the Republic of Kosovo.

Additional file 2: Figure S1. Phlebovirus seroprevalence by municipality in the Republic of Kosovo. Hatched lines indicate that no samples were available for this municipality. Overall seroprevalence (a), TOSV (b), and SFSV (c).

## Data Availability

Data are provided within the manuscript or supplementary information files.

## Abbreviations

TOSV: Toscana virus
SFSV: Sand fly fever Sicilian virus
SFNV: Sand fly fever Naples virus
ZVL: Zoonotic visceral leishmaniasis
CI: Confidence intervals
OR: Odds ratio
SFTSV: Severe fever with thrombocytopenia virus
RVFV: Rift Valley fever virus
NT-Ab: Neutralizing antibodies
NT: Neutralization
