# Supplementary material for: Neutralization-based seroprevalence of Toscana virus and sandfly fever Sicilian virus in dogs in the Republic of Kosovo
Source: Parasit Vectors. 2025 Feb 10;18:48. doi: 10.1186/s13071-025-06681-7 (PMC11812177; doi:10.1186/s13071-025-06681-7)
Supplement: Supplementary file 1 — Additional file 1: Table S1. Phlebovirus seroprevalence by municipality in the Republic of Kosovo. [file 13071_2025_6681_MOESM1_ESM.docx]

**Supplementary Table 1.** Phlebovirus seroprevalence by municipality.

|  |  |  | **total** | |  | **TOSV** | |  | **SFSV** | |
| --- | --- | --- | --- | --- | --- | --- | --- | --- | --- | --- |
| **District** | **Municipality** |  | **pos** | **%** |  | **pos** | **%** |  | **pos** | **%** |
| Prishtina (01) | Gllogoc (n=6) |  | 1 | 16.7 |  | 0 | 0.0 |  | 1 | 16.7 |
| Prishtina (01) | Gracanice (n=8) |  | 0 | 0.0 |  | 0 | 0.0 |  | 0 | 0.0 |
| Prishtina (01) | Kosove (n=6) |  | 1 | 16.7 |  | 1 | 16.7 |  | 0 | 0.0 |
| Prishtina (01) | Obiliq (n=14) |  | 1 | 7.1 |  | 1 | 7.1 |  | 0 | 0.0 |
| Prishtina (01) | Podujeve (n=6) |  | 0 | 0.0 |  | 0 | 0.0 |  | 0 | 0.0 |
| Prishtina (01) | Prishtine (n=10) |  | 5 | 50.0 |  | 5 | 50.0 |  | 0 | 0.0 |
| Mitrovica (02) | Leposavic (n=2) |  | 1 | 50.0 |  | 1 | 50.0 |  | 0 | 0.0 |
| Mitrovica (02) | Mitrovice (n=13) |  | 3 | 23.1 |  | 2 | 15.4 |  | 1 | 7.7 |
| Mitrovica (02) | Skenderaj (n=13) |  | 1 | 7.7 |  | 1 | 7.7 |  | 0 | 0.0 |
| Mitrovica (02) | Vushtrri (n=12) |  | 3 | 25.0 |  | 3 | 25.0 |  | 0 | 0.0 |
| Peja (03) | Istog (n=12) |  | 0 | 0.0 |  | 0 | 0.0 |  | 0 | 0.0 |
| Peja (03) | Peje (n=20) |  | 6 | 30.0 |  | 6 | 30.0 |  | 0 | 0.0 |
| Peja (03) | Kline (n=6) |  | 2 | 33.3 |  | 1 | 16.7 |  | 1 | 16.7 |
| Prizreni (04) | Dragash (n=8) |  | 0 | 0.0 |  | 0 | 0.0 |  | 0 | 0.0 |
| Prizreni (04) | Prizren (n=19) |  | 5 | 26.3 |  | 5 | 26.3 |  | 0 | 0.0 |
| Prizreni (04) | Suhareke (n=13) |  | 0 | 0.0 |  | 0 | 0.0 |  | 0 | 0.0 |
| Ferizaj (05) | Elezit (n=6) |  | 0 | 0.0 |  | 0 | 0.0 |  | 0 | 0.0 |
| Ferizaj (05) | Ferizaj (n=19) |  | 1 | 5.3 |  | 1 | 5.3 |  | 0 | 0.0 |
| Ferizaj (05) | Kaqanik (n=6) |  | 3 | 50.0 |  | 3 | 50.0 |  | 1 | 16.7 |
| Ferizaj (05) | Shterpce (n=5) |  | 1 | 20.0 |  | 0 | 0.0 |  | 1 | 20.0 |
| Ferizaj (05) | Shtime (n=5) |  | 0 | 0.0 |  | 0 | 0.0 |  | 0 | 0.0 |
| Gjilani (06) | Gjilan (n=8) |  | 0 | 0.0 |  | 0 | 0.0 |  | 0 | 0.0 |
| Gjilani (06) | Kamenic (n=14) |  | 1 | 7.1 |  | 0 | 0.0 |  | 1 | 7.1 |
| Gjilani (06) | Kllokot (n=2) |  | 1 | 50.0 |  | 1 | 50.0 |  | 0 | 0.0 |
| Gjilani (06) | Viti (n=16) |  | 2 | 12.5 |  | 2 | 12.5 |  | 0 | 0.0 |
| Gjakova (07) | Decan (n=2) |  | 1 | 50.0 |  | 0 | 0.0 |  | 1 | 50.0 |
| Gjakova (07) | Junik (n=7) |  | 1 | 14.3 |  | 0 | 0.0 |  | 1 | 14.3 |
| Gjakova (07) | Gjakove (n=13) |  | 3 | 23.1 |  | 2 | 15.4 |  | 2 | 15.4 |
| Gjakova (07) | Rahovec (n=17) |  | 2 | 11.8 |  | 1 | 5.9 |  | 1 | 5.9 |
