# Supplementary figures and images for: Neutralization-based seroprevalence of Toscana virus and sandfly fever Sicilian virus in dogs in the Republic of Kosovo
Source: Parasit Vectors. 2025 Feb 10;18:48. doi: 10.1186/s13071-025-06681-7 (PMC11812177; doi:10.1186/s13071-025-06681-7)

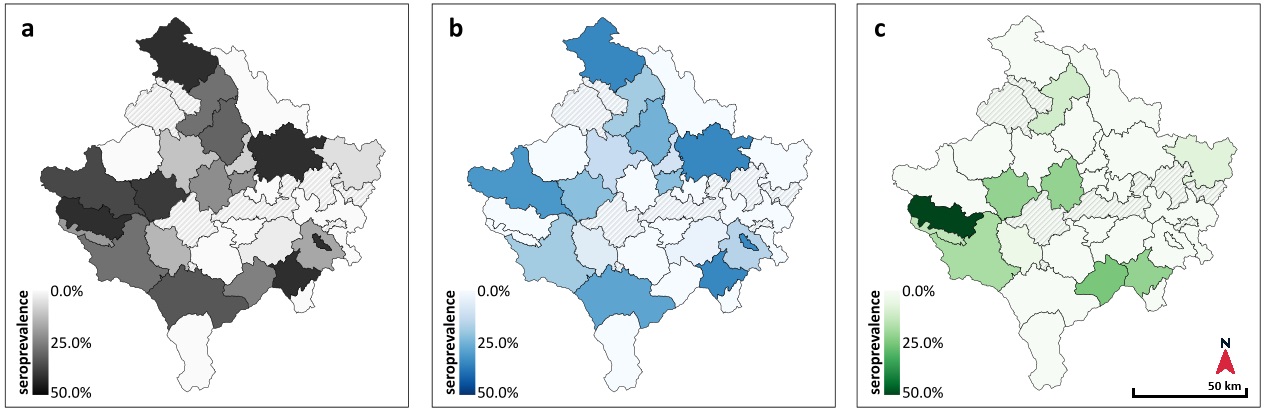

Supplement: Supplementary file 2 — Additional file 2: Figure S1. Phlebovirus seroprevalence by municipality in the Republic of Kosovo. Hatched lines indicate that no samples were available for this municipality. Overall seroprevalence (a), TOSV (b), and SFSV (c). [file 13071_2025_6681_MOESM2_ESM.jpg]
